# Premarital sexual practice and associated factors among high school youths in Debretabor town, South Gondar zone, North West Ethiopia, 2017

**DOI:** 10.1186/s13104-019-4348-3

**Published:** 2019-06-03

**Authors:** Wondmnew Lakew Arega, Taye Abuhay Zewale, Kassawmar Angaw Bogale

**Affiliations:** 1 Department of Reproductive Health, School of Public Health, College of Medicine and Health Sciences, Bahir-Dar University, Bahir Dar, Ethiopia; 2 Department of Epidemiology and Biostatistics, School of Public Health, College of Medicine and Health Sciences, Bahir-Dar University, Bahir Dar, Ethiopia; 3 Department of Epidemiology and Biostatistics, School of Public Health, College of Medicine and Health Sciences, Bahir-Dar University, Bahir Dar, Ethiopia

**Keywords:** Premarital sex, Youths, Debretabor, Ethiopia

## Abstract

**Objective:**

Premarital sex is voluntary sexual intercourse between unmarried persons. Prevalence and factors associated with premarital sexual practice in the study area are lacking. Thus, the aims of this study were to determine the prevalence and to identify factors associated with premarital sexual practice among Debretabor high school youths.

**Results:**

The prevalence of premarital sex among Debretabor town high school youths was 22.5% of which 63.9% of them were males. Among those high school youths, the majority (60.2%) had their first sexual intercourse at the age of 15–19 years. The main reason for initiation of sexual intercourse was due to fell in love which accounts 48.1%, followed by sexual desire 22.2%. Predictors that are risk for premarital sex were youths who did not attend religious education [AOR = 7.4, 95% CI (3.32, 16.43)], having boy or girl friends [AOR = 9.66, 95% CI (4.80, 19.43)], drinking alcohol every day [AOR = 9.43, 95% CI (2.86, 31.14)] and less than twice a week [AOR = 2.52, 95% CI (1.22, 5.21)], watching pornography film [AOR = 5.15, 95% CI (2.56, 10.37)] and youths came from rural residing families [AOR = 0.51, 95% CI (0.27, 0.96)].

## Introduction

Youths are in a state of rapid physical and psychological change. They have curiosity and urge to experience new phenomena [1]. Nevertheless, youths are exposed to different circumstance like fears, worries and different desires, they feel shame to get advice and guidance from their parents and elders [2]. Over a life cycle approach, youths and their communities need to know about reproductive health so that, they can make informed decisions about their reproductive health and sexuality [3, 4]. Premarital sex, defined as voluntary sexual intercourse between unmarried persons, is increasing worldwide [5]. It is unsafe because, most youths have no enough awareness on how to prevent and how to get guidance services on reproductive anatomy, physiology, sexually transmitted infection (STI), and Human Immunodeficiency Virus (HIV) [6, 7]. As a result, they are exposed to serious problems including premarital sex with its consequences and emotional scar [8, 9].

Though, schools are institutions where sufficient information and formal educations are provided to youths, premarital sexual practice among high school youths have been increased worldwide [10]. Globally, 35.3 million people live with HIV/AIDS of which youths account 2.1 million. Among 2.3 million new HIV infections, youths (15–24 years) account more than half [7].

Illegal abortions, risk of HIV infections and school dropout are the bad consequences of pre-marital sex in sub-Saharan Africa [11]. Up to 25% of 15–19 years, old youth’s exercised sex before age 15. In Ethiopia, the prevalence of premarital sex is increasing [12–14]. A study conducted in Eastern part of Ethiopia and Lalibela Town reported that above one-fourth of the school youths were exposed to premarital sex [12, 15]. Another study which is done in west Shoa Zone reported that about 60% of high school youths were exercised premarital sex [9]. Different scholars identified inconsistent factors which were positively or negatively associated with premarital sexual practice. Some of these factors includes age of students, sex, residence, educational level, peer pressure, having pocket money, substance use, alcohol drink, watching pornography movie, living arrangement, discussion with parents about sexual issues, having peers who are experienced sex and fall in love and access religious and life skill education [9, 12–17].

According to Debretabor district health office report sexually transmitted diseases, abortion and unwanted pregnancy are high in the study area among youths [18]. However, prevalence of premarital sexual practice and its associated factors among high school youths (grade 9th to grade 12th) in the study area was not dealt yet. Thus, this study aimed to determine premarital sexual practices and associated factors among high school youths in Debretabor town, south Gondar, Ethiopia.

## Main text

### Methods

#### Study design and setting

School based cross-sectional study design was conducted from September 18 to October 16, 2017, among high school youths in Debretabor town, South Gondar zone, Ethiopia. Debretabor town is located at 667 km from Addis Ababa, capital city of Ethiopia and has three high schools. The total numbers of high school youths in the study area by the year 2017 were 8892 (5220 females and 3672 males) [18].

#### Source population

The source population was all high school youths who were residing in Debretabor town and its surrounding rural Kebeles.

#### Study population

The study population was all high-school youths aged 15 to 24 years that were enrolled as a regular day-time student in 2017.

#### Inclusion criteria

All secondary school youths aged 15–24 attending regular class in Debretabor town during data collection period were included in the study.

#### Exclusion criterion

Married high school youths were excluded.

#### Sample size determination

Sample size was calculated using a single population proportion formula designated as ${\text{n}} = \frac{{(Z_{\alpha /2)}^{2} p\left( {1 - p} \right)}}{{d^{2} }}$ based on the assumptions of *P*-value = 0.25 which was the proportion of premarital sex among in-school youths in Jimma [19], a 95% confidence level, 4% margin of error (d) and 10% non-response rate. Accordingly, the total sample size calculated was 497.

#### Sampling procedure

All the high schools in the town were included in the study, and total sample size was proportionally allocated to each school. The lists of youths were obtained from the respective school registrar. Then, the study participants from each school were selected by computer generated simple random sampling technique after proportional allocation to their grade level.

#### Data collection

Pre-tested, self-administered structured Amharic (local language) questionnaire was used to collect the data. The questionnaire was pre-tested on 10% of the study participants at Alem-ber high school, which has the same setup to the study area, found in South Gondar zone. The questionnaire was amended according to the finding in the pretest before the distributions of final questionnaires. Training was given for data collectors and supervisors. Before the participants filled the questionnaires, the trained data collector gave orientation to youths regarding the aim of the study, the content of the questionnaire, the issue of confidentiality and respondents rights. Moreover, trained data collectors were involved in taking consents from participants and gathering filled questionnaires. However, data collectors were not present when the participants were filling the questionnaire.

The study used premarital sex practice as dependent variable and Socio-demographic Characteristics of youths and parents (age, sex, education level, religion, pocket money living arrangement, parental education, parental occupation, sexual issue discussion with parents), risk behavior and peer pressure (chat chewing, alcohol drinking, cigarette smoking, watching pornography, Peer friend initiation of sex) and history of partner hood, demand for condom utilization (number of partners, having the boy/girlfriend, condom utilization) as independent variable.

#### Data management and analysis

The data were entered using Epi-info version 7.2.1 and exported to SPSS version 23 for analysis. Descriptive statistics like frequency, percentage and standard deviation was computed. Binary logistics regression model was applied to identify determinant factors related premarital sexual practice. Variables with P value less than 0.25 on bi-variate analysis were entered to multi-variate analysis. 95% confidence interval was used to identify associated factors in multi-variable binary logistic regression model. Hosmer–Lemeshow goodness of the model fit was checked and analysis was done by entering procedure.

### Result

#### Socio-demographic characteristics of the respondents

Four hundred eighty high school youths were filled the questionnaire making a response rate of 96.6%. From the total respondents, more than half (53.8%) of them were females. Majority (71.2%) youths age were from 15 to 19 years. The average age and standard deviation of respondents were 17 and 1.29 years respectively. Larger proportion (35.8%) of the participants were grade nine students. The majority (97.5%) of the respondents were Orthodox Christians. Only 23.3% of in school youths had pocket money, about 89% of youths were living with their parents and attending religious services. Moreover, above half (61%) of the youths didn’t discuss about sexual issues with their parents (Table 1).

**Table 1:** Socio-demographic characteristics of high school youths in Debretabor town, South Gondar, Amhara Regional State, 2017

Variables	Frequency	Percent
Sex		
Male	222	46.2
Female	258	53.8
Residence		
Rural	239	49.8
Urban	241	50.2
Age		
13–17	342	71.2
18–24	138	28.8
Grade		
Grade 9th	172	35.8
Grade 10th	156	32.5
Grade 11th	90	18.8
Grade 12th	62	12.9
Religion		
Orthodox	468	97.5
Muslim	1	0.2
Protestant	6	1.3
Catholic	5	1.0
Do you attending religious services?		
No	53	11
Yes	427	89
Do you ever discussed about sexual issue with parents?		
Yes	187	39
No	293	61
Do you have pocket money?		
Yes	112	23.3
No	368	76.7
With whom do you live now?		
Alone	25	5.2
With parents	428	89.2
With friends/relatives	27	5.6

#### Sexual characteristics and risk behavior of respondents

From all respondents, 22.5% have had premarital sexual intercourse at the time of the survey, of which 63.9% were males and 60.2% had their first sexual intercourse at the age of 15–19 years. The main reason for initiation of sexual intercourse was due to fell in love which accounts 48.1%, followed by sexual desire 22.2%.

Concerning the number of sexual partners, majority (84.3%) of students have had sex with one partner and about 58% of the them were used condom during sexual intercourse. Coming to risky behavior, 28.1% of the youths drunk alcohol, 16.2% watched pornography and 2.7% chewed khat. About 61% of youths who watched pornography film were exposed to premarital sex (Table 2).

**Table 2:** Sexual history and risky behavior of high school youths in Debretabor town, South Gondar, Amhara regional State, 2017

Variable	Frequency	Percent
Did your peer friends experienced with sexual intercourse?		
Yes	56	11.7
No	424	88.3
Did your peer friend initiate you sexual intercourse?		
Yes	41	8.5
No	439	91.5
Have you ever chewing chat?		
Yes	13	2.7
No	467	97.3
Have you ever drink alcoholic beverage?		
Yes	135	28.1
No	345	71.9
Have you ever smoke cigarette?		
Yes	8	1.7
No	472	98.3
Have you ever watched sex film?		
Yes	78	16.2
No	402	83.8
Do you have boy/girl friend?		
Yes	108	22.5
No	372	77.5
Have you ever had premarital sexual intercourse?		
Yes	108	22.5
No	372	77.5
Reason to have sex		
Love	52	48.1
Sexual desire	24	22.2
Peer pressure	15	14.0
Chance	17	15.7
Did you use condom when you had sex (n = 108)?		
Yes	63	58.3
No	45	49.7
Did you use any contraceptive methods (n = 108)?		
Yes	44	40.7
No	64	67.3
Have you ever been pregnant (n = 39)?		
Yes	8	20.5
No	31	79.5
How many sexual partners do you have so far (n = 108)?		
One	91	84.3
Two	17	15.7

#### Factors associated with premarital sexual practice among high school youths in Debretabor town, 2017

The Logistic regression analysis showed that premarital sexual practice among youths who did not attend religious education was 7.4 times more likely exposed to premarital sex as compared to the counterpart [AOR = 7.4, 95% CI (3.32, 16.43)]. Similarly, youths who had a boy or a girl friend were 9.66 times more likely to start premarital sex than those who didn’t have a boy or a girl [AOR = 9.66, 95% CI (4.80, 19.43)]. Youths who were drinking alcohol every day and less than twice a week were 9.43 times [AOR = 9.43, 95% CI (2.86, 31.14)] and 2.52 times [AOR = 2.52, 95% CI (1.22, 5.21)] more likely engaged in premarital sex practice respectively as compared to those who did not drink alcohol. Students who watched pornography film were 5.15 times more likely practiced premarital sex as compared to those who didn’t watch pornography film [AOR = 5.15, 95% CI (2.56, 10.37)]. But it was found to be less likely among urban youths resident family as compared with youths who came from rural resident families (Table 3).

**Table 3:** Factors associated with premarital sex of high school youths in Debretabor town, South Gondar, 2017

Variable	Premarital sex		COR (95% CI)	AOR (95 CI)	P-value
	Yes	No			
Sex					
Male	153 (69%)	69 (21%)	1	1	
Female	219 (85%)	39 (15%)	0.39 (0.25, 0.61)	0.81 (0.43, 1.52)	0.51
Age					
15–19	273 (80%)	69 (20%)	1	1	
18–24	99 (72%)	39 (28%)	1.55 (0.98, 2.45)	1.39 (0.71, 2.70)	0.34
Residence					
Rural	172 (72%)	67 (28%)	1	1	
Urban	200 (83%)	41 (17%)	0.52 (0.33, 0.81)	0.51 (0.27, 0.96)	0.04
Live with whom					
Alone	13 (52%)	12 (48%)	1	1	
Parent	338 (79%)	90 (21%)	0.28 (0.12, 0.65)	0.44 (0.14, 1.37)	0.0.16
Friends	21 (78%)	6 (22%)	0.31 (0.09, 1.0)	0.73 (0.14, 3.78)	0.70
Pocket money					
Yes	73 (65%)	39 (35%)	1	1	
No	299 (81%)	69 (19%)	0.43 (0.27, 0.69)	0.82 (0.42, 1.62)	0.57
Attending religious education					
Yes	349 (82%)	78 (18%)	1	1	
No	23 (43%)	30 (57%)	5.83 (3.21, 10.59)	7.4 (3.32, 16.43)	0.00
Discussed about sexual issue with parents					
Yes	136 (73%)	51 (27%)	1	1	
No	236 (81%)	57 (18%)	0.64 (0.41, 0.99)	0.49 (0.20, 1.18)	0.115
Peer friend initiate you					
Yes	20 (49%)	21 (515)	1	1	1
No	352 (80%)	87 (20%)	0.23 (0.12, 0.45)	0.97 (0.2, 3.47)	0.969
Do you have boy/girl friend					
Yes	93 (43%)	124 (57%)		1	
No	15 (6%)	248 (94%)	12.40 (6.89, 22.28)	9.66 (4.80, 19.43)	0.000
Pornography watching					
Yes	44 (61%)	28 (39%)	9.13 (5.27, 15.81)	5.15 (2.56, 10.37)	0.00
No	59 (15%)	343 (85%)	1	1	
Have you ever drink alcoholic beverage					
Never	283 (85%)	49 (15%)	1	1	1
Less than twice a week	63 (66%)	32 (34%)	2.93 (1.74, 4.94)	2.52 (1.22, 5.21)	0.01
Twice a week	19 (59%)	13 (41%)	3.95 (1.83, 8.51)	2.67 (0.99, 7.21)	0.053
Everyday	7 (33%)	14 (67%)	11.55 (4.43, 30.06)	9.43 (2.86, 31.14)	0.000

### Discussion

Premarital sexual practice of high school youths in this study was 22.5% (CI: 19.0, 26.5). This finding was in line with a study conducted in Nekemtie town (21.5%) [20], in Jimma (21%) [19] and school youths in Alamata (21.1%) [21]. However, it was higher than in Coast Province, Kenya youths (14.9%) [22], and Robit high school youths (14.9%) [10]. The difference might be as a result of sample size, coast province; Kenya used existing data available from Kenya Global School Based Health Survey (GSHS) which was national study. So it could be more precise as compared with this study. In addition, there might be socio-cultural differences in community among study areas.

But this finding was lower than in-school youths in Ghana (42%) [23], in Jimma (28.5%) [24] in Eastern Ethiopia (24.8%) [12] and in Debremarkos high school youths (37.5% [25]. The variation may be due to difference in periods of the study (2011–2014), showing a changing and improving trend in easiness of reporting sexual matters and increasing premarital sexual awareness from time to time [7, 26]. The difference might also be due to variations on the prevalence of risky sexual behavior.

This study also found that those youths who didn’t attend religious education were more likely exposed to premarital sex as compared with their counter parts. It is in agreement with studies conducted in Bahir Dar City [14] and Mizan Aman [27]. The possible reason could be religious institutions strongly thought youths to be abstained until marriage.

High School youths who have a boyfriend or girlfriend were more likely to have premarital sexual intercourse than those who don’t. There were similar reports in Alamata [28], and Nekemt towns [20]. This could be due to the pressure from their boy/girl friend to have sexual practice.

Youths who drunk alcohol were engaged in premarital sexual practice as compared to their counterparts. This finding is the same as the studies done in South West [27] and Western Ethiopia [29]. The possible explanation might be, when youths drink alcohol, his/her ability of self-controlling decrease and this may expose to premarital sex.

Students who watched pornography film were more likely practiced premarital sex as compared to those who didn’t. There is similar finding in Shendi Town [13] and Northern Ethiopia [7]. The possible reason could be pornography film leads youths physiological and psychological motive for sexual intercourse.

Youths from rural family residents were more exposed to premarital sex urban youths. This is not in agreement with other studies [7, 21, 28, 30, 31]. This difference might be due to low parental control of rural youths as they lived with rented rooms, exposed to exercising sexual issues freely.

### Conclusions

Significant numbers of high school youths were engaged in sexual practice before marriage. Not attending religious education, have a boy/girlfriend, watching pornography film, alcohol drinkers and came from rural residing families were identified risk factors. So, the school community and respective health sector need to establish and strengthen school health program and school clubs to give awareness about identified risks of premarital sex. In addition family should link their youths to religious education in parallel to formal school education.

## Limitation of the study

Since the nature of this study is sensitive, reporting errors and biases can’t be controlled. In addition as this study used only quantitative data, the behavioral related information might be missed. Since the questionnaire was self-administered, lack of control over the responses rate, no control over who filled the questionnaires and questions may be miss-understood so that the true impression of the participants may not be gathered.

## Data Availability

All the data sets used for this study are available from the corresponding author and can be given with a reasonable request.

## Abbreviations

AIDS: Acquired Immune Deficiency Syndrome
AOR: adjusted odds ratio
CI: confidence interval
EC: Ethiopian calendar
DHS: Demographic Health Survey
EDHS: Ethiopia Demographic Health Survey
SD: standard deviation
BSC: Bachelor of Science
HIV: Human Immunodeficiency Virus
MPH: Masters of Public Health
SPSS: Statistical Package for Social Science
STI: sexually transmitted infection
WHO: World Health Organization
GSHS: Global School Based Health Survey
